# How long is too long: A retrospective study evaluating the impact of the duration of noninvasive oxygenation support strategies (high flow nasal cannula & BiPAP) on mortality in invasive mechanically ventilated patients with COVID-19

**DOI:** 10.1371/journal.pone.0281859

**Published:** 2023-02-16

**Authors:** Aditya Kasarabada, Kimberly Barker, Theresa Ganoe, Lindsay Clevenger, Cristina Visco, Jessica Gibson, Rahim Karimi, Negar Naderi, Brian Lam, Maria Stepanova, Linda Henry, Christopher King, Mehul Desai

**Affiliations:** 1 Medical Critical Care Service, Inova Fairfax Hospital, Falls Church, Virginia, United States of America; 2 Medicine Service Line, Inova Health Systems, Falls Church, Virginia, United States of America; 3 Department of Advanced Lung Disease and Transplant, Inova Fairfax Hospital, Falls Church, Virginia, United States of America; University of Palermo, ITALY

## Abstract

**Background/Aim:**

We investigated the association of noninvasive oxygenation support [high flow nasal cannula (HFNC) and BiPAP], timing of invasive mechanical ventilation (IMV), and inpatient mortality among patients hospitalized with COVID-19.

**Methods:**

Retrospective chart review study of patients hospitalized with COVID-19 (ICD-10 code U07.1) and received IMV from March 2020-October 2021. Charlson comorbidity index (CCI) was calculated; Obesity defined as body mass index (BMI) ≥ 30 kg/m^2^; morbid obesity was BMI ≥ 40 kg/m^2^. Clinical parameters/vital signs recorded at time of admission.

**Results:**

709 COVID-19 patients underwent IMV, predominantly admitted from March-May 2020 (45%), average age 62±15 years, 67% male, 37% Hispanic, and 9% from group living settings. 44% had obesity, 11% had morbid obesity, 55% had type II diabetes, 75% had hypertension, and average CCI was 3.65 (SD = 3.11). Crude mortality rate was 56%. Close linear association of age with inpatient-mortality risk was found [OR (95% CI) = 1.35 (1.27–1.44) per 5 years, p<0.0001)]. Patients who died after IMV received noninvasive oxygenation support significantly longer: 5.3 (8.0) vs. 2.7 (SD 4.6) days; longer use was also independently associated with a higher risk of inpatient-mortality: OR = 3.1 (1.8–5.4) for 3–7 days, 7.2 (3.8–13.7) for ≥8 days (reference: 1–2 days) (p<0.0001). The association magnitude varied between age groups: 3–7 days duration (ref: 1–2 days), OR = 4.8 (1.9–12.1) in ≥65 years old vs. 2.1 (1.0–4.6) in <65 years old. Higher mortality risk was associated with higher CCI in patients ≥65 (P = 0.0082); among younger patients, obesity (OR = 1.8 (1.0–3.2) or morbid obesity (OR = 2.8;1.4–5.9) (p<0.05) were associated. No mortality association was found for sex or race.

**Conclusion:**

Time spent on noninvasive oxygenation support [as defined by high flow nasal cannula (HFNC) and BiPAP] prior to IMV increased mortality risk. Research for the generalizability of our findings to other respiratory failure patient populations is needed.

## Introduction

SARS-CoV-2 is a novel respiratory coronavirus which can cause the infection now known as COVID-19 and can, in some cases, lead to death [1–7]. Among patients hospitalized with COVID-19, 20% progress to a severe disease state requiring invasive life support measures including invasive mechanical ventilation (IMV). However, when to initiate IMV among patients with ARDS has evolved over time.

At the beginning of the COVID-19 pandemic, patients were placed on IMV almost immediately. Questions then surfaced as to whether this mode of treatment was beneficial to patients or whether the use of noninvasive oxygenation support [as defined by high flow nasal cannula (HFNC) and BiPAP] should be considered as first-line treatment. As attention turned to the use of noninvasive oxygenation support strategies showing improved outcomes in prospective studies, other concerns were raised including the risk of infection transmission to healthcare workers through aerosolization [8]. In turn, these concerns lead to controversial recommendations provided by different scientific societies whereby noninvasive oxygenation support strategies in acute respiratory failure due to viral infection has been widely and variably used during the pandemic [8, 9].

Recently, a growing number of randomized controlled trials have tried to provide evidence on the effectiveness of noninvasive oxygenation support strategies in the management of COVID-19-associated acute hypoxemic respiratory failure [10–16]. However, these studies did not provide further guidance as to the duration of noninvasive oxygenation support methods before considering IMV. This is an especially important point as the work of breathing in those on noninvasive oxygenation support can cause significant damage to the pulmonary system such that using mechanical ventilation at a certain point may be futile among those who become intubated [8]. Therefore, the purpose of this study was to investigate the impact of the timing of IMV in conjunction with the use of noninvasive oxygenation support strategies [high flow nasal cannula (HFNC) and BiPAP] in patients who were hospitalized with COVID-19 and inpatient mortality.

## Methods

### Study design

This is a retrospective study. Data was obtained from our electronic health medical record (EMR) system (EPIC^®^) and was also retrieved from the health records using a case report form.

### Patient selection

All patients with a diagnosis of COVID-19 (ICD-10 code U07.1) who were hospitalized within Inova Health System, VA, USA between March 5^th^, 2020, and October 1^st^, 2021, received invasive mechanical ventilation (IMV) at any point during their admission and had a final discharge status at the time of the analysis were included. Each case was a unique case.

### Primary and secondary outcomes

The primary outcome of the study was inpatient mortality. The secondary outcome was to determine if there was a specific cut off period of time at which a patient receiving noninvasive oxygenation strategies [i.e. HFNC and/or BiPAP] should be considered for switching their respiratory support system to IMV.

### Study definitions

Using historic EMRs, we calculated the Charlson comorbidity index (CCI) for each patient using their medical history. Obesity was defined as a body mass index (BMI) ≥ 30 kg/m^2^, morbid obesity was defined as a BMI ≥ 40 kg/m^2^. Clinical parameters and vital signs were recorded at the time of admission during which mechanical ventilation occurred. Pre-intubation parameters included the number of days on noninvasive oxygenation support strategies before intubation which we defined as the number of days on BiPAP and/or the number of days spent on HFNC. We also collected other pertinent pre-intubation information which included: the use of vasodilators before intubation, Glasgow Coma Score (GCS) at intubation, and the PaO2/FiO2 at intubation.

### Statistical analysis

Patients’ parameters were summarized as N (%) or mean (±SD). Parameters were compared between groups using χ2 or Kruskal-Wallis tests for categorical or continuous parameters, respectively. Logistic regression was used to identify parameters associated with the study outcome. Two-sided p-values <0.05 were considered statistically significant.

SAS 9.4 (SAS Institute, Cary, NC) was used for all analyses.

The study was granted a waiver of consent and an exemption status by the Inova Health System’s Institutional Review Board #U20-04-4025 given that all data were deidentified and analyzed anonymously.

## Results

There were 709 COVID-19 patients who underwent mechanical ventilation treatment and were included in this study. Patients were predominantly admitted during the first months of the pandemic (45% in March-May 2020), were, on average, 62 (SD = 15) years of age with 12% younger than 45, 67% male, 23% white, 17% black, 37% Hispanic, 15% Asian, and 9% from group living setting (nursing home, long-term care, rehabilitation facilities). Of the included patients, 44% had obesity, 11% had morbid obesity, 55% had documented history of type 2 diabetes, 75% had a history of hypertension, and average CCI calculated from pre-COVID-19 medical history was 3.65 (SD = 3.11).

**Fig 1:**
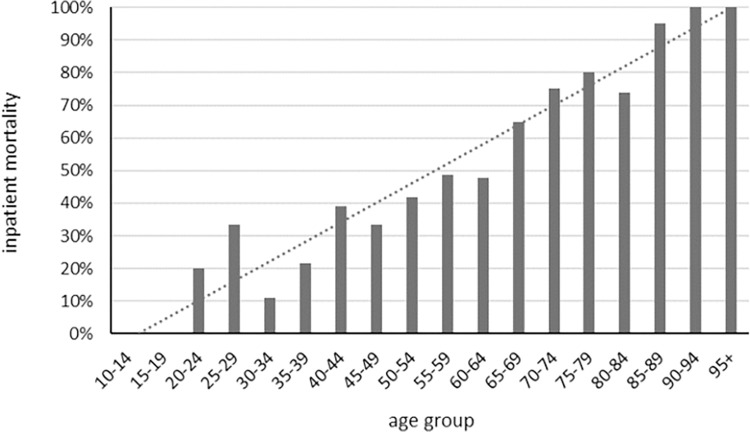
Linear association of inpatient mortality and age for COVID-19 patients on mechanical ventilation.

The crude mortality rate for COVID-19 patients on IMV was 56% (**Table 1**). Of those who were discharged alive, 54% were discharged home and 38% to a long-term care facility. In comparison to those discharged alive, COVID-19 patients who died after being intubated were, on average, 11 years older, equally likely male or female, less commonly Hispanic, more commonly from group living settings and had a higher CCI (p<0.01) (Table 1). In fact, there was a linear association of age with the risk of inpatient mortality for intubated patients with COVID-19 (OR (95% CI) = 1.35 (1.27–1.44) per 5 years, p<0.0001); that rate exceeded 50% for patients ≥65 years of age (Fig 1). In contrast, patients who were discharged alive incurred a longer length of inpatient stay and a higher rate of Extracorporeal membrane oxygenation (ECMO) utilization (p<0.01) (Table 1).

**Table 1:** Comparison of patients who died vs. not died on mechanical ventilation.

	Died	Not died	p	All
N	397 (56.0%)	312 (44.0%)		709
**Admission period:**				
Peak 1: March-May 2020	161 (40.6%)	157 (50.3%)	0.0094	318 (44.9%)
Plateau 1: June-October 2020	51 (12.8%)	45 (14.4%)	0.54	96 (13.5%)
Peak 2: November 2020-January 2021	141 (35.5%)	61 (19.6%)	< .0001	202 (28.5%)
Plateau 2: February-September 2021	44 (11.1%)	49 (15.7%)	0.07	93 (13.1%)
Age, years	66.6 ± 13.2	55.3 ± 14.1	< .0001	61.6 ± 14.7
Male	263 (66.2%)	211 (67.6%)	0.70	474 (66.9%)
Non-Hispanic white	100 (25.6%)	60 (19.4%)	0.0491	160 (22.9%)
Black or African-American	72 (18.5%)	44 (14.2%)	0.13	116 (16.6%)
Hispanic	127 (32.4%)	134 (43.1%)	0.0036	261 (37.1%)
Asian	53 (13.6%)	50 (16.1%)	0.35	103 (14.7%)
Other race/ethnicity	38 (9.7%)	22 (7.1%)	0.21	60 (8.6%)
Admitted from group living ^a^	50 (12.6%)	17 (5.4%)	0.0012	67 (9.4%)
BMI, kg/m2	30.7 ± 8.4	30.2 ± 8.0	0.36	30.5 ± 8.2
Obesity (BMI > 30)	173 (45.1%)	128 (42.1%)	0.44	301 (43.8%)
Morbid obesity (BMI > 40)	49 (12.8%)	28 (9.2%)	0.14	77 (11.2%)
Type 2 diabetes	231 (58.2%)	157 (50.3%)	0.0367	388 (54.7%)
Hypertension	314 (79.1%)	218 (69.9%)	0.0049	532 (75.0%)
Cirrhosis	15 (3.8%)	5 (1.6%)	0.08	20 (2.8%)
Charlson’s comorbidity index (CCI)	4.43 ± 3.22	2.66 ± 2.64	< .0001	3.65 ± 3.11
**Healthcare resource utilization:**				
Length of inpatient stay, days ^b^	19.9 ± 15.7	30.8 ± 20.8	< .0001	24.7 ± 18.9
Placed on ECMO	24 (6.0%)	45 (14.4%)	0.0002	69 (9.7%)
Had inpatient hospice status ^c^	43 (10.8%)	2 (0.6%)	< .0001	45 (6.3%)
**Pre-intubation parameters:**				
Received HFNC	233 (64.5%)	154 (57.5%)	0.07	387 (61.5%)
Received BiPAP	143 (41.0%)	57 (21.7%)	< .0001	200 (32.7%)
Total # days on noninvasive oxygenation strategies	5.27 ± 8.04	2.68 ± 4.65	< .0001	4.17 ± 6.91
# days on HFNC	3.62 ± 5.45	2.03 ± 3.49	0.0002	2.94 ± 4.78
# days on BiPAP	1.76 ± 5.02	0.77 ± 2.31	< .0001	1.33 ± 4.11
Vasodilators before intubation	75 (19.9%)	50 (16.9%)	0.33	125 (18.6%)
Glasgow coma score (GCS) at intubation	12.1 ± 4.2	12.8 ± 3.8	0.0033	12.4 ± 4.1
Initial peak pressure, mmHg	27.8 ± 8.1	26.9 ± 7.0	0.08	27.4 ± 7.6
Plateau peak pressure, mmHg	25.4 ± 6.8	24.2 ± 6.1	0.0197	24.8 ± 6.5
PaO2/ FiO2 at intubation	126.3 ± 86.6	154.8 ± 97.8	< .0001	138.5 +/- 92.6

^a^ group living includes those admitted from nursing home, long-term care facility, etc

^b^ it is the total length of inpatient stay including outside of ICU

^c^ the proportion of those who eventually moved to that status before discharged or died. ECMO- extracorporeal membrane oxygenation; HFNC- high flow nasal cannula; BiPAP-Bilevel positive airway pressure; PaO2- partial pressure of oxygen in the arterial blood; FIO2-fraction of inspired oxygen; BMI- body mass index

Considering pre-intubation parameters, patients who eventually died after intubation had received noninvasive oxygenation support strategies for a substantially longer period: mean (SD) 5.3 (8.0) vs. 2.7 (SD 4.6) days; that included longer duration of both nasal canula and BiPAP methods (p<0.01) (Table 1). Those patients also had lower GCS and PaO2/FiO2 ratio at intubation (p<0.01) although there was no difference in the rates of vasodilators use (p>0.05) (Table 1).

The crude mortality rate for IMV patients with COVID-19 was the highest during the winter of 2020–2021: 70% vs. 47–53% in other periods (p<0.0001) (S1 Table). That period was also associated with the highest mean age (65 vs. 62 overall) and CCI (3.9 vs. 3.6 overall) as well as the longest duration of pre-intubation respiratory support (mean 6.3 days vs. 4.2 days overall), the highest rate of BiPAP utilization (49% vs. 33% overall), and the lowest PaO2/FiO2 ratio (mean 125 vs. 139 overall) (p<0.01) (S1 Table).

**Fig 2:**
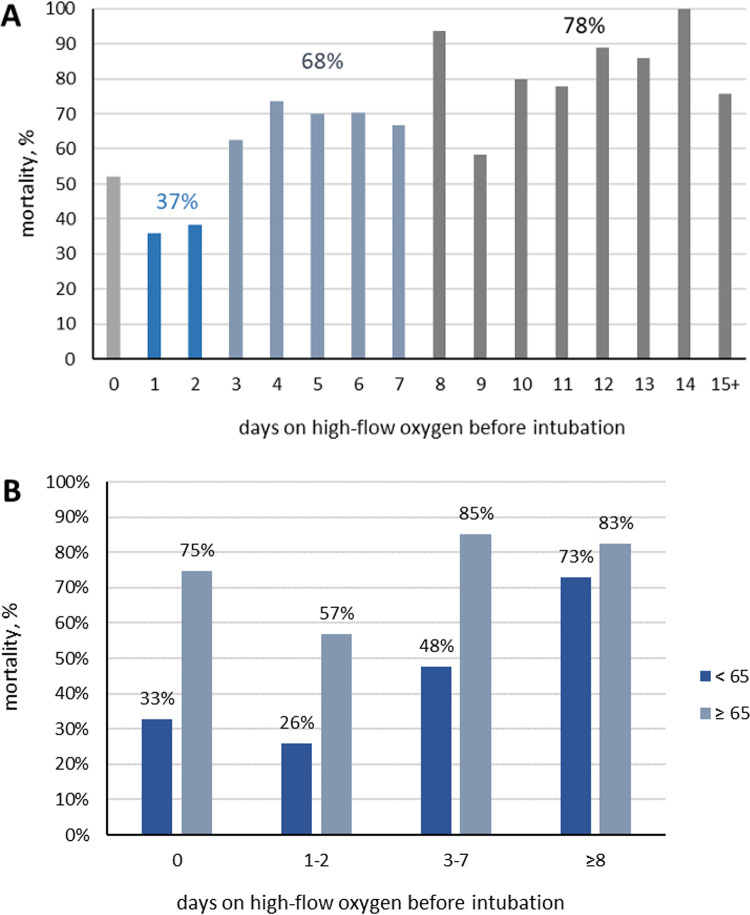
Inpatient mortality was higher with longer use of high flow oxygenation before intubation. (A) Inpatient mortality vs days on noninvasive oxygenation support differed across time intervals: Lowest for 1–2 days, higher for 3–7 days, and highest for 8+ days. (B) Inpatient mortality vs days on noninvasive oxygenation support stratified into 2 age groups: Age < 65 years and age ≥ 65 years. For younger group, differences in mortality for 1–2 days, 3–7 days, and 8+ days. For older group, differences in mortality in 1–2 days and 3+ days only.

Since there was a strong association between the duration of noninvasive oxygenation support strategies and post-intubation mortality (Table 1), we further assessed this association (Fig 2A). As a result, we found that patients who received noninvasive oxygenation support treatment (HFNC and BiPAP combined) for only 1 or 2 days had the lowest mortality rate of 37%, but that rate increased to 68% for patients who received noninvasive oxygenation support for 3–7 days and further increased to 78% for patients who had been on noninvasive oxygenation support for 8 or more days (Fig 2A, S2 Table). Finally, patients who had received no noninvasive oxygenation support [<1 day; zero days = mechanically intubated on admission] had a mortality rate of 52% (Fig 2A, S2 Table).

Since patients who had received noninvasive oxygenation support for only 1–2 days were the youngest of all (mean age 58 years vs. 64 years for patients who had received it for 3 days or longer), we additionally assessed the association of duration of noninvasive oxygenation support with the outcome stratified by age. As a result, we found that this association was indeed strongly mediated by age (Fig 2B). Specifically, patients in the <65 age group had a mortality rate of 26% if intubation followed 1–2 days on noninvasive oxygenation support; that rate increased to 48% for patients intubated after 3–7 days of noninvasive oxygenation support, and then to 73% for intubation after 8 or more days noninvasive oxygenation support (Fig 2B). In contrast, in patients of ≥65 years of age, the lowest mortality rate was 57% if intubated after 1–2 days on noninvasive oxygenation support; that rate increased to 83–85% for patients intubated after 3 or more days of noninvasive oxygenation support, without any additional association with the duration (Fig 2B).

In multivariate analysis, increased time on noninvasive oxygenation support was independently associated with a higher risk of post-intubation mortality: OR (95% CI) = 3.1 (1.8–5.4) for 3–7 days, 7.2 (3.8–13.7) for ≥8 days (reference: 1–2 days) (p<0.0001) **(Table 2).** As seen in the univariate analysis discussed above, the magnitude of this association was found to vary between age groups: for 3–7 days duration (ref: 1–2 days), OR = 4.8 (1.9–12.1) in ≥65 years old vs. 2.1 (1.0–4.6) in <65 years old; for ≥8 days duration (ref: 1–2 days), OR = 5.3 (2.0–13.9) in ≥65 years old vs. 8.6 (3.7–20.0) for <65 years old **(Table 3**).

**Table 2:** Independent predictors of inpatient mortality in COVID-19 patients on mechanical ventilation.

predictor	OR (95% CI)	p
Admission period (ref: off-peak periods)		
Peak 1 (March-May 2020)	1.49 (0.94–2.38)	0.09
Peak 2 (November 2020-January 2021)	2.37 (1.40–4.02)	0.0014
Age (ref: <45 years)		
45–54 years	0.76 (0.39–1.46)	0.41
55–64 years	1.02 (0.55–1.87)	0.96
65–74 years	1.96 (1.04–3.68)	0.0373
≥75 years	2.52 (1.19–5.34)	0.0157
Male sex (ref: female)	1.25 (0.83–1.88)	0.30
CCI (ref: 0–2)		
CCI = 3–4	1.69 (0.99–2.88)	0.05
CCI = 5–8	2.18 (1.26–3.78)	0.0053
CCI ≥ 9	4.13 (1.78–9.55)	0.0009
Obesity (BMI > 30)	1.54 (1.01–2.36)	0.0457
Morbid obesity (BMI > 40)	2.16 (1.13–4.14)	0.0198
Cirrhosis	6.47 (1.64–25.54)	0.0077
Total # days on noninvasive oxygenation support before intubation (ref: 1–2 days)		
0 days	1.63 (0.97–2.75)	0.07
3–7 days	3.09 (1.76–5.42)	< .0001
≥8 days	7.23 (3.82–13.67)	< .0001
GCS at intubation, per 1 point	0.94 (0.89–0.99)	0.0230

CCI- Charlson Comorbidity Scale; GCS- Glascow Coma Scale

**Table 3:** Independent predictors of inpatient mortality in COVID-19 patients on mechanical ventilation by age group.

period	OR (95% CI)	p
Age group: ≥ 65 years		
Admission period (ref: off-peak periods)		
Peak 1 (March-May 2020)	2.25 (1.06–4.79)	0.0352
Peak 2 (November 2020-January 2021)	3.07 (1.38–6.84)	0.0062
Age ≥ 75 (ref: age 65–74)	0.97 (0.49–1.91)	0.93
Male sex	0.99 (0.52–1.91)	0.98
CCI = 3–4 (ref: CCI ≤2)	2.37 (0.93–6.03)	0.07
CCI = 5–8 (ref: CCI ≤2)	2.81 (1.11–7.15)	0.0300
CCI ≥ 9 (ref: CCI ≤2)	4.59 (1.48–14.2)	0.0082
Obesity (BMI > 30)	1.29 (0.67–2.50)	0.45
Morbid obesity (BMI > 40)	0.94 (0.21–4.33)	0.94
Total # days on noninvasive oxygenation support prior to intubation (ref: 1–2 days)		
0 days	1.86 (0.82–4.25)	0.14
3–7 days	4.79 (1.90–12.08)	0.0009
≥8 days	5.27 (2.00–13.91)	0.0008
GCS at intubation, per 1 point	0.92 (0.85–1.01)	0.07
Age group: <65 years		
Admission period (ref: off-peak periods)		
Peak 1 (March-May 2020)	1.06 (0.57–1.99)	0.86
Peak 2 (November 2020-January 2021)	1.76 (0.84–3.69)	0.13
Age 55–64 (ref: age <55)	1.84 (1.06–3.19)	0.0294
Male sex	1.58 (0.88–2.84)	0.13
CCI = 3–4 (ref: CCI ≤2)	1.12 (0.54–2.30)	0.76
CCI = 5–8 (ref: CCI ≤2)	1.03 (0.44–2.43)	0.94
CCI ≥ 9 (ref: CCI ≤2)	1.42 (0.23–8.66)	0.70
Obesity (BMI > 30)	1.76 (0.97–3.20)	0.06
Morbid obesity (BMI > 40)	2.83 (1.35–5.94)	0.0061
Cirrhosis	8.32 (1.9–36.52)	0.0050
Total # days on noninvasive oxygenation support before intubation (ref: 1–2 days)		
0 days	1.37 (0.67–2.79)	0.39
3–7 days	2.15 (1.01–4.60)	0.0483
≥8 days	8.6 (3.71–19.97)	< .0001
GCS at intubation, per 1 point	0.98 (0.91–1.06)	0.61

CCI- Charlson Comorbidity Scale; GCS- Glascow Coma Scale; BMI-Body Mass Index

Other independent predictors of post-intubation mortality in COVID-19 also varied between the age groups. Specifically, in the older age group, the association of the outcome with the period of admission was significant; patients ≥65 were more likely to die if admitted during local peaks of infections and hospitalizations (spring 2020 and winter 2020–2021): OR = 2.25 (1.06–4.79) and 3.07 (1.38–6.84), respectively (both p<0.05); in contrast, there was no such association for patients <65 (both p>0.05) (Table 3). The association of a relatively older age with mortality was observed in the <65 group only (55–64 vs. <55) while association with higher CCI was increasingly significant only in patients ≥65 (Table 3). At the same time, younger patients were marginally at higher risk of mortality if obese (OR = 1.76 (0.97–3.20, P = 0.06) but significantly at risk if morbidly obese (OR = 2.8 (1.4–5.9, p<0.01) while no such association was found in the older age group (p>0.05) (Table 3). Finally, after adjustment for these factors, no association of post-intubation mortality with sex or race was found in either age group (all p>0.05).

## Discussion

Although IMV can be a life-saving treatment for those with COVID-19, we found that among patients with severe ARDS, the timing of when to initiate this mode of ventilation is vital, especially for those 65 years and older. As noted in our analysis of over 700 patients with COVID-19 who received IMV, the use of noninvasive oxygenation support strategies for greater than seven days increased the mortality substantially, regardless of a patient’s prior comorbidity status.

However, on closer observation of the data, the effect of noninvasive oxygenation support strategies was mediated by age. Patients 65 years and older saw a substantial increase in mortality if they were on this modality for more than two days before being intubated and mechanically ventilated. In contrast, patients who were 64 years and younger had up to seven days in which they could receive noninvasive oxygenation support before seeing a substantial increase in mortality. It is worth noting that regardless, the mortality risk in this patient population also began to increase after two days on this modality. Our finding is somewhat different than an earlier report which indicated that those who received IMV within their first two days had a higher mortality than those who were intubated later in their hospitalization [17]. However, the authors did find that for those who eventually needed IMV, their mortality was higher which is in line with our results. The investigators, as well, determined that the exact timing of the use of IMV in those with COVID-19 induced respiratory failure is very complex as we note in this study [17].

Such a finding does make sense, as the work of breathing with high-flow oxygen can cause significant lung and airway damage to the point that the use of mechanical ventilation to deliver oxygen is no longer a viable option [18]. In recent studies, respiratory efforts among some patients with COVID-19 who were breathing spontaneously have been shown to cause significant lung damage similar to ventilator-induced lung injury from high pressures- a situation known as self-inflicted lung injury (P-SILI) [18–20]. We suggest that the use of noninvasive oxygenation support in those who meet severe ARDS criteria be carefully considered when weighing each oxygenation system’s known risks and benefits.

Another interesting finding from this study is that the use of BiPAP significantly differed between patients who died and those who survived. Patients who died received approximately one-day longer of BiPAP compared to those who did not die. This was not the aim of our study; however, this finding is in line with a recent study that demonstrated that the use of a high-flow nasal cannula may provide better survival from mechanical ventilation than BiPAP [21, 22]. These patients also appeared to have already received a longer duration of HFNC prior to being placed on BiPAP, so we suggest that the combined duration may be affecting the outcome. Further study is warranted to determine the best noninvasive support delivery device to use (i.e. HFNC vs BiPAP).

We also noted that the other predictors for mortality differed by age. In patients 65 years and older, the greater the number of comorbidities present on admission for COVID-19, the higher the risk of death. Admission during the peaks of COVID-19 was also associated with a higher risk of death. For patients younger than 65, neither the comorbidity burden nor being admitted during the peaks of COVID-19 were significant risk factors. However, morbid obesity (BMI ≥ 40) increased the risk of dying three-fold.

Morbid obesity has been identified as a predictor of mortality in other studies [23, 24]. Our study highlights its impact on those under the age of 65 regardless of other comorbidities present, which helps to confirm a prior study where morbid obesity was also found to be a significant risk factor for those 50 years and younger [25]. Studies on the interaction of obesity and COVID-19 continue; it is hypothesized that patients with obesity have impaired immune responses and abnormal secretion of proinflammatory cytokines such as interleukin 6 (IL-6), which are already present due to COVID-19, worsening the disease. Morbid obesity may also cause a decrease in the lungs’ functional residual capacity, causing hypoxemia, while the expression of angiotensin-converting enzyme 2 (ACE2) from the adipose tissue also has a high affinity to SARS-CoV-2, which may increase the virality of COVID-19 [26–30].

The increased mortality during the two peaks of COVID-19 highlighted in this study can partially be explained by the current respiratory management principles at each corresponding time point. During the first peak, we used IMV on most patients per the recommendations before studies found that IMV should not be the first line of treatment. As such, the second peak captures the use of the recommended noninvasive oxygenation support prior to IMV. However, as noted in our study, the length of time on noninvasive oxygenation support may have been detrimental, whereby attention to the time on high flow for patients who continue in respiratory distress despite therapy should be considered when deciding on the use of IMV.

These latter points are essential even though COVID-19 has evolved and changed in its lethality, partially due to new treatments and the availability of vaccines to ward off severe illness. However, in the United States, COVID-19 is still active; the reported 7-day daily average for COVID-19-related hospitalizations is close to 4000 (Sept 2022) with almost 400 COVID-19-related deaths reported as a 7-day moving average [31]. It is disturbing to note that patients 65 years and older now make up over 50% of hospitalizations, which was not the case previously. Therefore, we suggest that given our findings, if noninvasive oxygenation support is considered a treatment option, careful thought is still warranted when determining how much time should be spent on noninvasive oxygenation support prior to IMV among the sickest patients.

Furthermore, more research is needed on the use of, and length of time spent on noninvasive oxygenation support among patients who develop ARDS for reasons outside of COVID-19 to determine if our findings are valid in another patient population. This suggestion is vital given prior studies that reported similar results to ours for those with acute respiratory failure [32, 33]. A recent study conducted among cancer patients requiring intensive oxygen therapy for acute respiratory failure found that a longer duration of noninvasive oxygenation support use (3 days or more) before intubation increased mortality risk by almost 8 times [33].

## Limitations

There are several limitations to the study. This is a retrospective, single health system review of data which may limit the generalizability of the results. There is a potential for selection bias as the outcome of mechanical ventilation was the inclusion criteria for this cohort. Patient selection for mechanical ventilation in COVID-19 changed as the pandemic evolved, leading to differing approaches for noninvasive oxygenation support prior to intubation. As mentioned previously, early in the pandemic, patients were intubated and placed on mechanical ventilation early in the time course of the disease. During the second wave, more patients were placed on noninvasive methods of oxygenation and for longer durations prior to intubation for IMV. Nevertheless, we accounted for each wave of COVID-19 in our multivariate analysis, and time spent on noninvasive oxygenation support remained significant, suggesting that despite a change in guidelines, the length of time spent on noninvasive oxygenation support prior to IMV increased the risk of mortality. Data were abstracted from chart review, which depends on the strength and quality of the original documentation.

## Conclusion

As the COVID-19 pandemic continues to evolve, so does our understanding of treating those who experience ARDS. In this study, we found that among those who received IMV, the most significant predictor for in-hospital mortality was the length of time spent on noninvasive oxygenation support prior to IMV, such that the more time spent on noninvasive oxygenation support, the higher the odds of dying. However, the effect of time spent on noninvasive oxygenation support was mediated by age. Patients 65 years and older experienced an increase in mortality after spending only 2 days on noninvasive oxygenation support while those 64 years and younger experienced an incremental increase in mortality risk up to 7 days where by day 7 the risk of mortality increased significantly. While these results require further study, we suggest that careful consideration be given to the use of noninvasive oxygenation support versus IMV keeping in mind the patient’s age and other risk factors. In addition, further research is needed to determine the generalizability of these results to other patient populations with acute respiratory failure from different etiologies.

## Supporting information

S1 Table Comparison of patients on mechanical ventilation by the period of admission. (DOCX)

S2 Table Comparison of patients by the number of days on high-flow oxygen before intubation. (DOCX)
