# Supplementary material for: How long is too long: A retrospective study evaluating the impact of the duration of noninvasive oxygenation support strategies (high flow nasal cannula & BiPAP) on mortality in invasive mechanically ventilated patients with COVID-19
Source: PLoS One. 2023 Feb 16;18(2):e0281859. doi: 10.1371/journal.pone.0281859 (PMC9934441; doi:10.1371/journal.pone.0281859)
Supplement: S1 Table — (DOCX) [file pone.0281859.s001.docx]

**S1 Table**. Comparison of patients on mechanical ventilation by the period of admission.

|  | Peak 1: March-  May 2020 | Summer 2020: Jun-Oct | Peak 2: Nov  2020-Jan 2021 | Post-peak 2021: Feb-Sept | p |
| --- | --- | --- | --- | --- | --- |
| N | 318 | 96 | 202 | 93 |  |
| Age, years | 60.5 ± 14.7 | 62.6 ± 14.0 | 64.9 ± 14.7 | 57.3 ± 14.4 | 0.0001 |
| Male | 218 (68.6%) | 62 (64.6%) | 133 (65.8%) | 61 (65.6%) | 0.85 |
| Non-Hispanic white | 59 (18.7%) | 19 (20.0%) | 55 (27.6%) | 27 (30.0%) | 0.0326 |
| Black or African-American | 58 (18.4%) | 19 (20.0%) | 25 (12.6%) | 14 (15.6%) | 0.27 |
| Hispanic | 140 (44.2%) | 37 (38.5%) | 62 (31.0%) | 22 (24.4%) | 0.0010 |
| Asian | 43 (13.6%) | 11 (11.6%) | 37 (18.6%) | 12 (13.3%) | 0.31 |
| Other race/ethnicity | 16 (5.1%) | 9 (9.5%) | 20 (10.1%) | 15 (16.7%) | 0.0043 |
| Admitted from group living | 39 (12.3%) | 15 (15.6%) | 13 (6.4%) | 0 (0.0%) | 0.0003 |
| BMI, kg/m2 | 30.3 ± 7.9 | 30.4 ± 7.5 | 29.6 ± 8.0 | 32.7 ± 9.9 | 0.0462 |
| Obesity (BMI > 30) | 132 (43.6%) | 42 (43.8%) | 77 (39.1%) | 50 (54.3%) | 0.11 |
| Morbid obesity (BMI > 40) | 36 (11.9%) | 10 (10.4%) | 15 (7.6%) | 16 (17.4%) | 0.10 |
| Type 2 diabetes | 168 (52.8%) | 60 (62.5%) | 111 (55.0%) | 49 (52.7%) | 0.40 |
| Hypertension | 230 (72.3%) | 79 (82.3%) | 154 (76.2%) | 69 (74.2%) | 0.25 |
| Cirrhosis | 11 (3.5%) | 1 (1.0%) | 6 (3.0%) | 2 (2.2%) | 0.63 |
| Charlson's comorbidity index |  |  |  |  |  |
| (CCI) | 3.57 ± 3.13 | 3.76 ± 3.37 | 3.94 ± 2.97 | 3.22 ± 3.01 | 0.10 |
| **Discharge:** |  |  |  |  |  |
| Died | 161 (50.6%) | 51 (53.1%) | 141 (69.8%) | 44 (47.3%) | 0.0001 |
| Home (including home |  |  |  |  |  |
| healthcare) | 104 (32.7%) | 18 (18.8%) | 24 (11.9%) | 23 (24.7%) | <.0001 |
| Hospice (including home |  |  |  |  |  |
| hospice) | 3 (0.9%) | 2 (2.1%) | 5 (2.5%) | 1 (1.1%) | 0.53 |
| Long-term care facility | 47 (14.8%) | 23 (24.0%) | 30 (14.9%) | 20 (21.5%) | 0.09 |
| Short-term care facility | 3 (0.9%) | 2 (2.1%) | 2 (1.0%) | 5 (5.4%) | 0.0250 |
| **Healthcare resource** |  |  |  |  |  |
| **utilization:** |  |  |  |  |  |
| Length of inpatient stay, days | 22.9 ± 16.6 | 27.8 ± 21.1 | 24.9 ± 19.1 | 27.4 ± 23.0 | 0.22 |
| Placed on ECMO | 34 (10.7%) | 8 (8.3%) | 15 (7.4%) | 12 (12.9%) | 0.42 |
| Had inpatient hospice status | 23 (7.2%) | 6 (6.3%) | 13 (6.4%) | 3 (3.2%) | 0.58 |
| **Pre-intubation parameters:** |  |  |  |  |  |
| Number/Percent who received HFNC  Number/Percent who received | 163 (56.8%) | 52 (62.7%) | 119 (68.4%) | 53 (62.4%) | 0.10 |
| BiPap | 48 (17.2%) | 32 (39.0%) | 82 (49.4%) | 38 (44.7%) | <.0001 |
| Total # days on noninvasive |  |  |  |  |  |
| Oxygenation support | 2.47 ± 4.65 | 4.62 ± 5.58 | 6.30 ± 9.81 | 5.13 ± 6.17 | <.0001 |
| # days on HFNC | 2.01 ± 3.96 | 2.76 ± 3.60 | 4.29 ± 5.91 | 3.52 ± 5.06 | 0.0002 |
| # days on BiPAP | 0.498 ± 1.655 | 1.89 ± 3.45 | 2.31 ± 6.70 | 1.61 ± 3.09 | <.0001 |
| Vasodilators before intubation | 46 (15.1%) | 21 (23.9%) | 35 (18.2%) | 23 (26.4%) | 0.05 |
| Glasgow coma score (GCS) at  intubation | 12.7 ± 3.9 | 12.2 ± 4.1 | 12.0 ± 4.3 | 12.5 ± 4.1 | 0.10 |
| Initial peak pressure, mmHg | 26.8 ± 7.1 | 27.7 ± 7.7 | 28.0 ± 8.8 | 28.1 ± 6.6 | 0.19 |
| Plateau peak pressure, mmHg | 23.7 ± 5.8 | 24.2 ± 5.7 | 26.0 ± 7.3 | 27.0 ± 7.1 | 0.0002 |
| PaO2/FiO2 at intubation | 145.7 ± 91.1 | 137.6 ± 92.2 | 125.4 ± 92.7 | 142.3 ± 96.6 | 0.0025 |

^a^ group living includes those admitted from nursing home, long-term care facility, etc.^b^ it is the total length of inpatient stay including outside of ICU, ^c^ the proportion of those who eventually moved to that status before discharged or died. ECMO- extracorporeal membrane oxygenation; HFNC- high flow nasal cannula; BiPAP-Bilevel positive airway pressure; PaO2- partial pressure of oxygen in the arterial blood; FIO2-fraction of inspired oxygen; BMI- body mass index
