# Supplementary material for: How long is too long: A retrospective study evaluating the impact of the duration of noninvasive oxygenation support strategies (high flow nasal cannula & BiPAP) on mortality in invasive mechanically ventilated patients with COVID-19
Source: PLoS One. 2023 Feb 16;18(2):e0281859. doi: 10.1371/journal.pone.0281859 (PMC9934441; doi:10.1371/journal.pone.0281859)
Supplement: S2 Table — (DOCX) [file pone.0281859.s002.docx]

**S2 Table**. Comparison of patients by the number of days on high-flow oxygen before intubation.

|  | 0 days | 1-2 days | | 3-7 days | | | ≥8 days | | p | |
| --- | --- | --- | --- | --- | --- | --- | --- | --- | --- | --- |
| N | 205 | 144 | | 137 | | | 122 | |  | |
| Age, years | 61.6 ± 16.2 | 57.7 ± 15.9 | | 64.0 ± 13.7 | | | 63.6 ± 11.4 | | 0.0021 | |
| Age <45 | 30 (14.6%) | 28 (19.4%) | | 12 (8.8%) | | | 6 (4.9%) | | 0.0015 | |
| Age 45-54 | 34 (16.6%) | 30 (20.8%) | | 23 (16.8%) | | | 23 (18.9%) | | 0.74 | |
| Age 55-64 | 46 (22.4%) | 35 (24.3%) | | 28 (20.4%) | | | 30 (24.6%) | | 0.84 | |
| Age 65-74 | 48 (23.4%) | 28 (19.4%) | | 39 (28.5%) | | | 38 (31.1%) | | 0.11 | |
| Age 75-84 | 31 (15.1%) | 18 (12.5%) | | 32 (23.4%) | | | 24 (19.7%) | | 0.07 | |
| Age 85-94 | 15 (7.3%) | 5 (3.5%) | | 3 (2.2%) | | | 1 (0.8%) | | 0.0148 | |
| Age ≥95 | 1 (0.5%) | 0 (0.0%) | | 0 (0.0%) | | | 0 (0.0%) | | 0.58 | |
| Male | 139 (67.8%) | 88 (61.1%) | | 95 (69.3%) | | | 79 (64.8%) | | 0.46 | |
| Non-Hispanic white | 49 (24.5%) | 23 (16.0%) | | 33 (24.3%) | | | 23 (19.0%) | | 0.19 | |
| Black or African-American | 44 (22.0%) | 24 (16.7%) | | 21 (15.4%) | | | 15 (12.4%) | | 0.14 | |
| Hispanic | 63 (31.2%) | 57 (39.6%) | | 54 (39.4%) | | | 53 (43.8%) | | 0.11 | |
| Asian | 29 (14.5%) | 26 (18.1%) | | 20 (14.7%) | | | 14 (11.6%) | | 0.53 | |
| Other race/ethnicity | 15 (7.5%) | 14 (9.7%) | | 8 (5.9%) | | | 16 (13.2%) | | 0.18 | |
| Admitted from group living | 31 (15.1%) | 13 (9.0%) | | 12 (8.8%) | | | 1 (0.8%) | | 0.0003 | |
| BMI, kg/m2 | 29.7 ± 9.0 | 31.7 ± 9.3 | | 30.8 ± 7.4 | | | 30.5 ± 6.5 | | 0.10 | |
| Obesity (BMI > 30) | 80 (40.8%) | 71 (51.4%) | | 60 (45.1%) | | | 55 (45.5%) | | 0.30 | |
| Morbid obesity (BMI > 40) | 17 (8.7%) | 22 (15.9%) | | 19 (14.3%) | | | 10 (8.3%) | | 0.09 | |
| Type 2 diabetes | 101 (49.3%) | 72 (50.0%) | | 86 (62.8%) | | | 83 (68.0%) | | 0.0014 | |
| Hypertension | 158 (77.1%) | 104 (72.2%) | | 106 (77.4%) | | | 89 (73.0%) | | 0.63 | |
| Cirrhosis | 5 (2.4%) | 5 (3.5%) | | 5 (3.6%) | | | 2 (1.6%) | | 0.73 | |
| Charlson's comorbidity index |  |  | |  | | |  | |  | |
| (CCI) | 4.04 ± 3.46 | 3.22 ± 2.88 | | 4.01 ± 3.18 | | | 3.47 ± 2.85 | | 0.09 | |
| **Discharge:** |  |  | |  | | |  | |  | |
| Died | 107 (52.2%) | 53 (36.8%) | | 93 (67.9%) | | | 95 (77.9%) | | <.0001 | |
| Home (including home healthcare) | 64 (31.2%) | 52 (36.1%) | | 18 (13.1%) | | | 5 (4.1%) | | <.0001 | |
| Hospice (including home hospice) | 3 (1.5%) | 2 (1.4%) | | 4 (2.9%) | | | 1 (0.8%) | | 0.57 | |
| Long-term care facility | 30 (14.6%) | 36 (25.0%) | | 19 (13.9%) | | | 15 (12.3%) | | 0.0158 | |
| Short-term care facility | 1 (0.5%) | 1 (0.7%) | | 3 (2.2%) | | | 6 (4.9%) | | 0.0206 | |
| **Healthcare resource utilization:** |  |  | |  | | |  | |  | |
| Length of inpatient stay, days | 18.4 ± 19.0 | 24.8 ± 15.6 | | 25.6 ± 15.9 | | | 34.7 ± 21.9 | | <.0001 | |
| Placed on ECMO | 14 (6.8%) | 23 (16.0%) | | 14 (10.2%) | | | 12 (9.8%) | | 0.05 | |
| Had inpatient hospice status | 22 (10.7%) | 5 (3.5%) | | 10 (7.3%) | | | 3 (2.5%) | | 0.0093 | |
| **Pre-intubation parameters:** |  |  | |  | | |  | |  | |
| Number/precent who received HFNC | 0 (0.0%) | 123 (85.4%) | | 126 (92.0%) | | | 118 (96.7%) | | <.0001 | |
| Number/percent received BiPAP | 0 (0.0%) | 37 (25.7%) | | 74 (54.0%) | | | 86 (70.5%) | | <.0001 | |
| Total # days on noninvasive oxygenation support | 0.000 ± 0.000 | 1.33 ± 0.47 | | 4.78 ± 1.44 | | | 13.8 ± 10.2 | | <.0001 | |
| # days on HFNC | 0.000 ± 0.000 | | 1.04 ± 0.58 | | 3.23 ± 1.92 | 9.49 ± 6.66 | | <.0001 | |  |
| # days on BiPAP | 0.000 ± 0.000 | 0.285 ± 0.511 | | 1.55 ± 1.86 | | | 4.34 ± 7.80 | | <.0001 | |
| Vasodilators before intubation | 2 (1.0%) | 28 (20.0%) | | 39 (29.1%) | | | 46 (39.0%) | | <.0001 | |

| Glasgow coma score (GCS) at intubation | 10.8 ± 4.9 | 13.5 ± 3.2 | 13.2 ± 3.0 | 13.5 ± 3.1 | <.0001 |
| --- | --- | --- | --- | --- | --- |
| Initial peak pressure, mmHg | 26.0 ± 6.8 | 28.2 ± 6.7 | 28.7 ± 8.2 | 30.2 ± 7.5 | <.0001 |
| Plateau peak pressure, mmHg | 23.2 ± 5.8 | 24.3 ± 5.3 | 25.5 ± 7.1 | 29.1 ± 5.7 | <.0001 |
| PaO2/FiO2 at intubation **Vital signs and laboratory parameters at admission:** | 176.9 ± 112.8 | 138.0 ± 83.9 | 115.4 ± 68.1 | 99.5 ± 54.9 | <.0001 |
| Diastolic blood pressure, mmHg | 68.1 ± 17.3 | 71.7 ± 16.5 | 69.5 ± 13.6 | 70.8 ± 15.0 | 0.33 |
| Systolic blood pressure, mmHg | 127.0 ± 30.6 | 130.1 ± 29.2 | 123.8 ± 28.2 | 127.4 ± 25.7 | 0.64 |
| Temperature, F | 98.9 ± 2.2 | 99.2 ± 1.8 | 99.0 ± 1.8 | 98.8 ± 1.9 | 0.71 |
| Heart rate, per min | 92.4 ± 24.5 | 96.6 ± 24.0 | 87.2 ± 26.7 | 87.1 ± 26.8 | 0.0094 |
| Respiration rate, per min | 23.8 ± 8.2 | 26.7 ± 10.2 | 26.6 ± 12.6 | 27.1 ± 12.8 | 0.0026 |
| Oxygen saturation, % | 89.4 ± 13.6 | 86.3 ± 13.2 | 88.1 ± 10.4 | 84.8 ± 12.5 | <.0001 |
| Saturation ≤90% | 64 (31.2%) | 71 (49.3%) | 65 (47.4%) | 70 (57.4%) | <.0001 |
| ALT, U/L | 65.1 ± 151.3 | 51.3 ± 49.3 | 49.1 ± 48.5 | 46.0 ± 35.0 | 0.84 |
| AST, U/L | 90.4 ± 178.7 | 76.3 ± 101.9 | 65.2 ± 55.4 | 63.6 ± 43.2 | 0.75 |
| Bicarbonate, mEq/L | 21.1 ± 5.4 | 22.3 ± 5.6 | 21.1 ± 4.2 | 22.3 ± 4.4 | 0.09 |
| Creatinine, mg/dL | 2.14 ± 2.85 | 1.69 ± 2.33 | 1.62 ± 1.52 | 1.63 ± 2.03 | 0.0154 |
| C-reactive protein, mg/L | 14.6 ± 11.3 | 17.0 ± 10.5 | 14.3 ± 8.7 | 15.5 ± 10.1 | 0.08 |
| D-dimer, ng/mL | 5.39 ± 11.72 | 2.86 ± 4.45 | 2.22 ± 3.32 | 2.32 ± 3.45 | <.0001 |
| Hemoglobin, g/dL | 12.7 ± 3.2 | 12.8 ± 2.2 | 12.7 ± 2.4 | 13.5 ± 2.2 | 0.0016 |
| Lymphocyte count, 10^3/mL | 1.19 ± 1.23 | 1.04 ± 0.97 | 0.967 ± 0.822 | 0.896 ± 0.984 | 0.0109 |
| Platelet count, 10^9/L | 211.0 ± 102.6 | 222.8 ± 95.0 | 213.7 ± 91.1 | 214.1 ± 82.7 | 0.66 |
| Total bilirubin, mg/dL | 0.872 ± 1.854 | 0.799 ± 1.668 | 0.734 ± 0.696 | 0.626 ± 0.360 | 0.89 |
| White blood count, 10^3/uL | 9.96 ± 5.87 | 9.66 ± 5.91 | 8.70 ± 4.76 | 8.17 ± 4.79 | 0.0122 |

^a^ group living includes those admitted from nursing home, long-term care facility, etc.^b^ it is the total length of inpatient stay including outside of ICU, ^c^ the proportion of those who eventually moved to that status before discharged or died. ECMO- extracorporeal membrane oxygenation; HFNC- high flow nasal cannula; BiPAP-Bilevel positive airway pressure; PaO2- partial pressure of oxygen in the arterial blood; FIO2-fraction of inspired oxygen; BMI- body mass index
